# A Novel Wind Speed Estimation Based on the Integration of an Artificial Neural Network and a Particle Filter Using BeiDou GEO Reflectometry

**DOI:** 10.3390/s18103350

**Published:** 2018-10-08

**Authors:** Kittipong Kasantikul, Dongkai Yang, Qiang Wang, Aung Lwin

**Affiliations:** 1 Doctoral Program on Space Technology Applications, Beijing 100191, China; 2 School of Electronic and Information Engineering, Beihang University, Beijing 100191, China; edkyang@buaa.edu.cn (D.Y.); wangqiang_620@163.com (Q.W.); uaunglwin@ieee.org (A.L.)

**Keywords:** wind speed estimation, GNSS-reflectometry, artificial neural network, particle swarm optimization, particle filter, BeiDou GEO satellite

## Abstract

Oceanographic remote sensing, which is based on the sensitivity of reflected signals from the Global Navigation Satellite Systems (GNSS), so-called GNSS-Reflectometry (GNSS-R), is very useful for the observation of ocean wind speed. Wind speed estimation over the ocean is the core factor in maritime transportation management and the study of climate change. The main concept of the GNSS-R technique is using the different times between the reflected and the direct signals to measure the wind speed and wind direction. Accordingly, this research proposes a novel technique for wind speed estimation involving the integration of an artificial neural network and the particle filter based on a theoretical model. Moreover, particle swarm optimization was applied to find the optimal weight and bias of the artificial neural network, in order to improve the accuracy of the estimation result. The observation dataset of the reflected signal information from BeiDou Geostationary Earth Orbit (GEO) satellite number 4 was used as an input for the estimation model. The data consisted of two phases with *I* and *Q* components. Two periods of BeiDou data were selected, the first period was from 3 to 8 August 2013 and the second period was from 12 to 14 August 2013, which corresponded to events from the typhoon Utor. The in situ wind speed measurement collected from the buoy station was used to validate the results. A coastal experiment was conducted at the Yangjiang site located in the South China Sea. The results show the ability of the proposed technique to estimate wind speed with a root mean square error of approximately 1.9 m/s.

## 1. Introduction

Global Navigation Satellite Systems (GNSS) technologies are widely used for positioning, navigation, and time applications based on information from direct signals. In addition, the reflected signals from satellites over the land or ocean surface can be applied for Earth observations known as GNSS-reflectometry (GNSS-R). This is a well-established technique for remote sensing that is used in many applications to observe the geophysical parameters of the Earth. The first bistatic radar remote sensing technique was proposed for an ocean altimetry application, based on the L-band of Global Positioning System (GPS) signal, in 1993 by the European Space Agency [1]. Several platforms have been widely discussed and developed to capture GNSS-R information, such as space-borne, satellite, and ground stations. It is highly effective for monitoring the Earth’s environments, measuring significant wave heights, wind speeds, soil moisture, and ice and snow thicknesses [2,3,4,5]. In particular, the characteristics of wind speed are an important factor for the observations of typhoon and hurricane events [6,7]. In 2003, Surrey Satellite Technology developed the first GPS-R receiver onboard the UK-Disaster Monitoring Constellation (UK-DMC) satellite in order to establish ocean and land surface conditions [8]. Recently, the Cyclone Global Navigation Satellite System (CYGNSS) project was developed by the National Aeronautics and Space Administration (NASA) to predict the occurrences and observe the characteristics of hurricanes. CYGNSS consists of eight micro-satellites launched into a low Earth orbit with a 35 degree inclination. Each satellite receives information from four GPS satellites [9,10]. The main advantage of this technique is the large number of navigation satellites in space, such as the GPS, GLONASS, Galileo, and BeiDou satellite constellations, which provide more information and a greater prominent spatial-temporal sampling capability over the Earth’s surface. Moreover, the benefit of long-term ubiquitous information that is freely available provides the opportunity to achieve global coverage at a lower cost compared to an active instrument.

**Figure 1:**
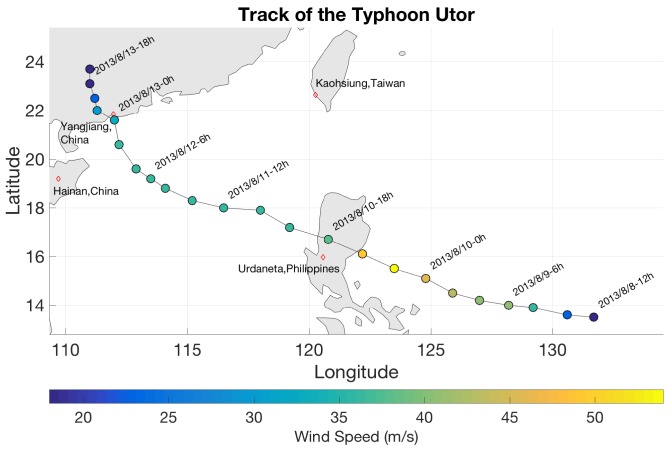
Map of typhoon Utor’s track. The minimum and maximum wind speeds were approximately 18 and 54 m/s, respectively.

The Chinese BeiDou navigation satellite system (BDS) has already provided excellent performance in terms of precise positioning, Total Electron Content (TEC) estimation, and modeling of the Earth’s ionosphere, etc. principally in the Asia-Pacific region [11,12]. As of July 2017, BeiDou consists of five Geostationary Earth Orbit (GEO), six Inclined Geo-Synchronization Orbiters (IGSO), and three Medium Earth Orbiters (MEO). Furthermore, by 2020, BeiDou constellations will fully support global full-time and full-weather, which can be applied in many applications, especially for short message communication services. In the same way, the information from reflected signals was studied in many remote sensing applications [13,14,15,16]. From 12 to 14 August 2013, the typhoon Utor formed in the Eastern part of the Philippines and dissipated in Southern China. Figure 1 shows the tracking map of the typhoon Utor simulated from wind speed information from the Japan Meteorological Agency. Consequently, during the typhoon period, this paper estimated wind speeds using the dataset from the BeiDou satellite.

In general, wind speed estimation based on reflected signals has been developed and discussed. For instance, Least Squares (LS) fitting of the measured Delay Doppler Map (DDM) to the theoretical Zavorotny and Voronvich model and the regression technique based on geophysical parameters. Both techniques have been widely applied and the results show good agreement with in situ wind speed measurement [17,18,19,20,21,22,23]. However, this technique does not learn the characteristics of the variables. For this reason, a machine learning technique was applied to estimate wind speeds [24]. In this work, we propose a combination of an artificial neural network (ANN) and a particle filter (PF) to estimate wind speeds based on the reflected signal information of the BeiDou G4 satellite. The study area focuses on the ocean surface of the South China Sea near the cost of the Yangjiang province. In addition, particle swarm optimization (PSO) was applied to find the optimal weight and bias of the ANN because of its high efficiency and easy implementation; it only requires the adjustment of a few parameters [25,26,27]. Moreover, the particle filter was implemented as an adaptive algorithm that is independent of missing factors and more robust in nature [28,29,30,31].

The remainder of this paper is organized as follows: Section 2 describes the study area and the specific details of the data collection; Section 3 and Section 4 present the algorithm for wind speed estimation, which includes the ANN, PSO, and PF techniques; and Section 5 discusses the error comparison and experimental results. Finally, the conclusions are presented in Section 6.

## 2. Data Description and Preprocessing

**Figure 2:**
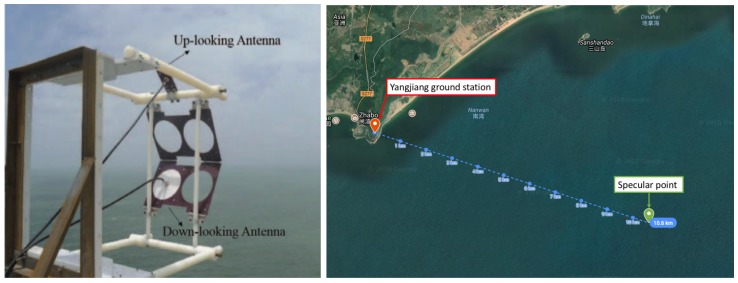
The ground receiving station at Yangjiang site to collect the reflected signal from the BeiDou G4 satellite. Up and down-looking antenna (**left**) and the distance between receiving station and specular point (**right**).

The raw intermediate frequency (IF) was captured from the up-and down-looking antennae by the dual-isolated GNSS radio frequency application specific integrated circuit (RF-ASIC) components, from which the antennae point to the ocean. The bandwidth of the captured IF signal for BeiDou G4 is approximately 4 MHz, centered at 3.996 MHz. Two-bit data were collected from both the direct and reflected signals. In order to illustrate the proposed technique for wind speed estimation, the observation data collected from 3 to 8 and 12 to 14 August 2013 at the Yangjiang ground station. Figure 2 shows the station site which located on the Eastern coast of China at 21°33′58.50″ N and 111°51′38.93″ E at a height of 120 m above the mean sea level. The station received the signal from the transmitter position of the BeiDou G4 satellite (0°0′0″ N, 160°0′0″ E) at a height of 35,801 km, and the specular point was approximately 21°32′11.4″ N, 111°57′28.08″ E. The reflected signal captured from the receiver station consisted of phase *I* and *Q* components, which are presented in the Experimental Results section.

## 3. Methodology

### 3.1. Artificial Neural Network

**Figure 3:**
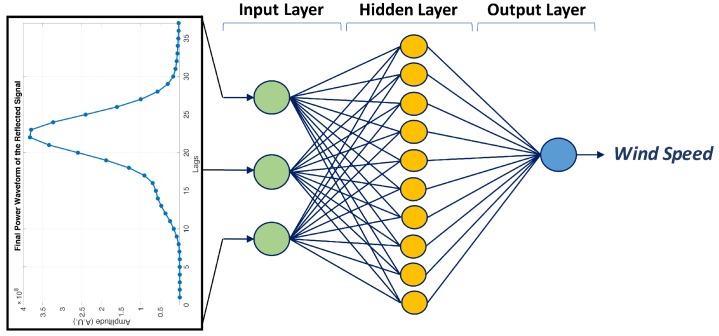
The architecture of a feed-forward ANN for wind speed estimation using the reflected signal from the BeiDou satellite.

Artificial neural networks (ANNs) are powerful computational models, inspired by the functions of biological neural networks, which were designed to simulate the behavior of the human neural system based on the relationships between input information and the target. ANN is a robust data modeling tool that is widely used in many applications, such as regression, classification, and approximation-based learning processes [32,33]. A common architecture of ANN is divided into three layers, consisting of the input layer, the hidden layer, and the output layer, as illustrated in Figure 3.

Each input element ($p=[p_{1},p_{2},p_{3},\ldots ,p_{n}]$) has a different weight ($w_{1},w_{2},w_{3},\ldots ,w_{n}$) for each neuron. The net input can be calculated as follows:

(1) $$n=\sum_{i=1}^{R}w_{ij}p_{j}+b=Wp+b,$$

where $w_{ij}$ is the corresponding connection weight between neuron (*i*) from the input layer and neuron (*j*) from the hidden layer, and *b* is the bias of the neuron. This research presents a Multilayer Perceptron (MLP) neural network including two layers of neurons by configuring ten artificial neurons for the hidden layer and one artificial neuron for the output layer. The hyperbolic tangent sigmoid activation function was applied to the hidden neurons and can be expressed by:

(2) $$f\left(n\right)=\frac{2}{1+e^{-2n}}-1.$$

Subsequently, a linear function was applied to the output layer of the ANN. The model was implemented using the two layer feed-forward neural network in which the weights and biases were trained by the particle swarm optimization algorithm, based on the input information and target; this is presented in the next section. In accordance with this algorithm, the error functions were updated through a number of iterations. The network learning error can be expressed by:

(3) $$E=\frac{1}{N}\sum_{i=1}^{N}{\left(y_{i}-t_{i}\right)}^{2},$$

where $y_{i}$ is the final output of the network, $t_{i}$ is the target, and *N* is the length of the output. The training process stopped when the conditions were met.

### 3.2. Particle Swarm Optimization

In 1995, Kennedy and Eberhart proposed a parallel algorithm called particle swarm optimization (PSO), which is a stochastic optimization technique that was inspired by the social behavior of fish schooling patterns [34]. The basic concept of PSO starts by initializing a group of random particles (solutions) and then searches for optimal parameter values by updating the generations in a *D*-dimensional solution search space. For each iteration, the particle updates itself based on tracking two parameters which consist of the personal best value $\left(P_{p\_best}\right)$ and the group best value $\left(P_{g\_best}\right)$. The target value is set as the best value of the fitness function. This new position is simply calculated as the sum of the previous position and the new velocity as follows:

(4) $$X_{i,t+1}=X_{i,t}+V_{i,t+1},$$

(5) $$V_{i,t+1}=\omega_{i}V_{i,t}+c_{1}r_{1}\left(P_{p\_best_{{}_{i,t}}}-X_{i,t}\right)+c_{2}r_{2},\left(P_{g\_best_{i,t}}-X_{i,t}\right),$$

where $X_{i,t}$ and $V_{i,t}$ are the position and velocity of the particle for iteration *t*, respectively. $\omega_{i}$ is the weight of the inertia factor, $c_{1}$ and $c_{2}$ are the acceleration coefficients, and $r_{1}$ and $r_{2}$ are random numbers in the range [0,1]. The weight inertia is designed to reduced linearly and can be expressed by:

(6) $$\omega_{i}=\omega_{max}-\frac{\omega_{max}-\omega_{min}}{iter_{max}}\times t,$$

where $\omega_{min}$ and $\omega_{max}$ are the minimum and maximum inertia weights. $iter_{max}$ is the maximum number of iterations. A summary of the PSO approach is shown in Algorithm 1.

**Algorithm 1:** Pseudocode for particle swarm optimization

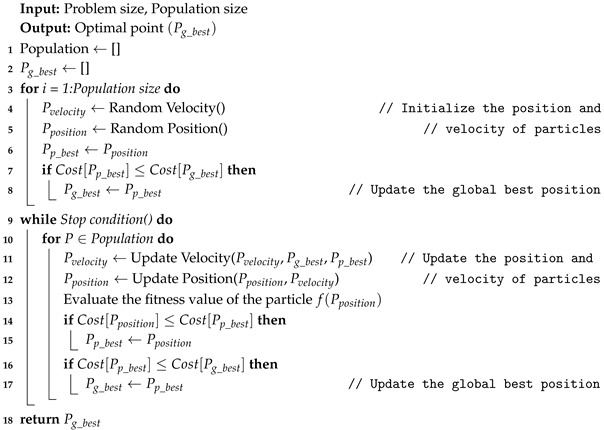



### 3.3. Coastal GNSS-Reflectometry Waveform Modeling

The theoretical scattering model based on the bistatic equation and Kirchhoff approximation was developed by Zavorotny and Voronvich and is known as the Z–V model which describes the averaged power waveform of the reflected signal consist of delay Δτ and Doppler frequency offset Δf with respect to the nominal specular point [35]. It is widely used to simulate the delayed waveform of the reflected signal from the satellite, which can be expressed by:

(7) $$|Y_{0}{\left(\tau \right)|}^{2}=\frac{T_{i}^{2}P_{t}G_{t}\lambda^{2}}{{\left(4\pi \right)}^{3}}\int \frac{G_{r}\left(\vec{r}\right)\Lambda^{2}\left(\tau \left(r\right)-\Delta \tau \right)S^{2}\left(T_{i}\left(f\left(r\right)-\Delta f\right)\right)}{R_{G}^{2}\left(r\right)R_{R}^{2}\left(r\right)}\sigma^{0}\left(r\right)dr,$$

where $\vec{r}$ is the spatial coordinate on the sea surface, and $T_{i}$ is the coherent integration time. $P_{t}$ is the transmitted power from the satellite, and λ is the signal wavelength. $G_{t}$ and $G_{r}$ are the gains of the transmit and receiver antenna, respectively. $R_{G}$ and $R_{R}$ are the ranges between the ocean surface point and the satellite and receiver, respectively, Λ(·) is the autocorrelation function of signal from the satellites. S(·) is the frequency modulation function. $\sigma^{0}\left(\cdot \right)$ is a bistatic radar cross section (BRCS) of the rough surface which depends upon the surface slope probability density function ($\left(P(\cdot )\right)$) and is expressed as:

(8) $$\sigma^{0}\left(r\right)=\pi {\left|ℜ\right|}^{2}\frac{q^{4}\left(r\right)}{q_{z}^{4}\left(r\right)}P\left(-\frac{q_{⊥}}{q_{z}}\right),$$

where *ℜ* is the Fresnel reflection coefficient. The Mean Square Slopes (MSS) of the up and cross winds ($\sigma_{u}$ and $\sigma_{c}$, respectively) are approximated based on the calibrated sea surface roughness model developed by Katzberg [36] and are calculated as follows:

(9) $$\begin{array}{c} \sigma_{u}=0.45\cdot \left[0.00+0.00316\cdot f\left(U\right)\right],\\ \sigma_{c}=0.45\cdot \left[0.003+0.00192\cdot f\left(U\right)\right], \end{array}$$

where f(U) is related to the in situ wind speed measurement (U) and can be calculated as follows:

(10) $$f\left(U\right)=\left\{\begin{array}{cc} U, & 0<U\leq 3.49,\\ 6\ln(U)-4.0, & 3.49<U\leq 46,\\ 0.411\;U, & 46<U. \end{array}\right.$$

This work focuses on the wind speed at the height of 10 m above the sea surface which is the default value for the anemometer height. The relationship of the wind speed measured at different heights [37] can be expressed as:

(11) $$U_{2}=U_{1}{\left(z_{2}/z_{1}\right)}^{P},$$

where $U_{2}$ is the wind speed at the desired reference height $z_{2}$, and $U_{1}$ is the wind speed measured at height $z_{1}$. A value for the exponent *P* is 0.11 was determined to be applicable most of the time over the ocean.

### 3.4. Particle Filter

The particle filter is a sequential Monte Carlo technique that is used to solve state estimation problems [38]. The basic idea is to represent the required posterior density function by a set of random samples with associated weights and then compute a new generation of samples based on these samples and weights. The construction of a discrete system dynamic model is mainly expressed by two equations and can be written as:

(12) $$x_{k}=F\left(x_{k-1}\right)+w_{k},$$

(13) $$z_{k}=H\left(x_{k-1}\right)+v_{k},$$

where $x_{k}$ is the system’s state at time *k*, F(·) is the system’s model, $x_{k-1}$ is the system’s state at the previous time, $z_{k}$ is the measurement state, H(·) is the measurement or observation model, and both $w_{k}$ and $v_{k}$ are noise. This paper used the autoregressive model (AR) to estimate the wind speed. The AR parameters were determined by using the previous iteration estimate information of the wind speed in an iteration particle filter system. The state-space representation of the AR model can be expressed by:

(14) $$\begin{array}{c} \left[\begin{array}{c} x_{k}^{i}\\ \varphi_{1}\\ \varphi_{2}\\ \varphi_{3}\\ x_{k-1}^{i}\\ x_{k-2}^{i}\\ x_{k-3}^{i} \end{array}\right]=\left[\begin{array}{ccccccc} \varphi_{1} & 0 & 0 & 0 & \varphi_{2} & \varphi_{3} & 0\\ 0 & 1 & 0 & 0 & 0 & 0 & 0\\ 0 & 0 & 1 & 0 & 0 & 0 & 0\\ 0 & 0 & 0 & 1 & 0 & 0 & 0\\ 1 & 0 & 0 & 0 & 0 & 0 & 0\\ 0 & 0 & 0 & 0 & 1 & 0 & 0\\ 0 & 0 & 0 & 0 & 0 & 1 & 0 \end{array}\right]\left[\begin{array}{c} x_{k-1}^{i}\\ \varphi_{1}\\ \varphi_{2}\\ \varphi_{3}\\ x_{k-2}^{i}\\ x_{k-3}^{i}\\ x_{k-4}^{i} \end{array}\right]+w_{k}, \end{array}$$

where *i* is the particle number and *k* is the sample time. $\varphi_{1}$, $\varphi_{2}$, and $\varphi_{3}$ are the autoregressive parameters. The measurement model was described in the previous section. The predicted distribution of $x_{k}$ is given by $p(x_{k}|z_{1:k-1})$, which can be expressed by:

(15) $$p\left(x_{k}|z_{1:k-1}\right)=\int p\left(x_{k}|x_{1:k-1}\right)p\left(x_{k}|z_{1:k-1}\right)dx_{k-1}.$$

**Figure 4:**
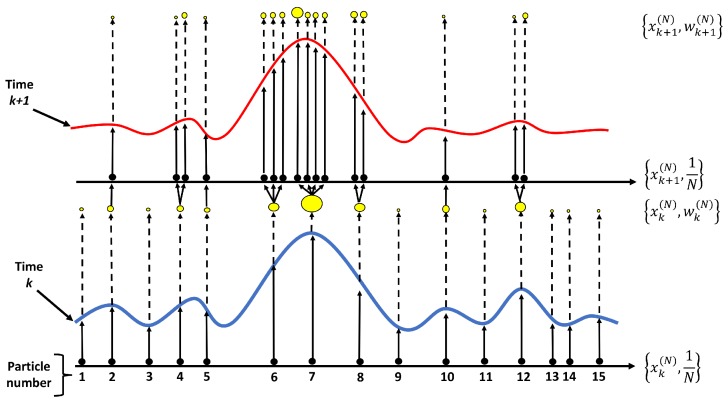
A schematic of the particle filter process.

For each time step, the normalization weight $\left(\sum w_{k}^{i}=1\right)$ and the posterior probability density distribution function can be approximated as follows:

(16) $$p\left(x_{1:k}|z_{1:k}\right)\approx \sum_{i=1}^{N}w_{k}^{i}\delta \left(x_{1:k}-x_{1:k}^{i}\right),$$

where:

(17) $$w_{k}^{i}\propto \frac{p(x_{k}^{i}|z_{1:k})}{q(x_{k}^{i}|z_{1:k})},$$

(18) $$p\left(x_{k}|z_{1:k}\right)=\frac{p\left(z_{k}|x_{k},z_{1:k-1}\right)p\left(x_{k}|z_{1:k-1}\right)}{p(z_{k}|z_{1:k-1})},$$

(19) $$q\left(x_{k}|z_{1:k}\right)=q\left(x_{k}|x_{k},z_{1:k-1}\right)q\left(x_{k-1}|z_{1:k-1}\right),$$

and q(·) is the proposal density. Afterwards, the weights are normalized in order to determine the importance of the sample based on the resampling technique. The final step is to generate a new generation of particles that are close to the measurement values. The particle filter process is illustrated in Figure 4.

## 4. Experimental Results

**Figure 5:**
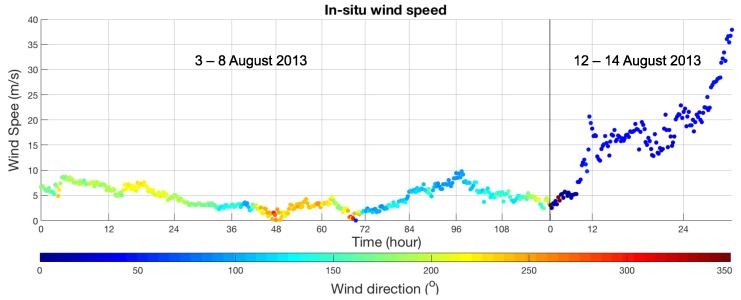
The in situ wind speed measurement from the buoy station from 3 to 8 August 2013 and 12 to 14 August 2013. The average wind speed of each time period are approximately 3.5 and 16 m/s, respectively.

The efficiency of the proposed technique for wind speed estimation based on the reflected signal is evaluated in this section. The observation dataset was collected during the typhoon Utor, which was a powerful tropical cyclone that formed in the Eastern part of the Philippines and dissipated in Southern China. The in situ wind speed measurement was collected from two periods, the first period was from 3 to 8 August 2013, in which the minimum and maximum wind speeds were 0.1 and 8.9 m/s, respectively. The second period was from 12 to 14 August 2013, in which the minimum and maximum wind speeds were 2.5 and 37.9 m/s, respectively. Figure 5 shows the wind speed changes over the two periods.

**Figure 6:**
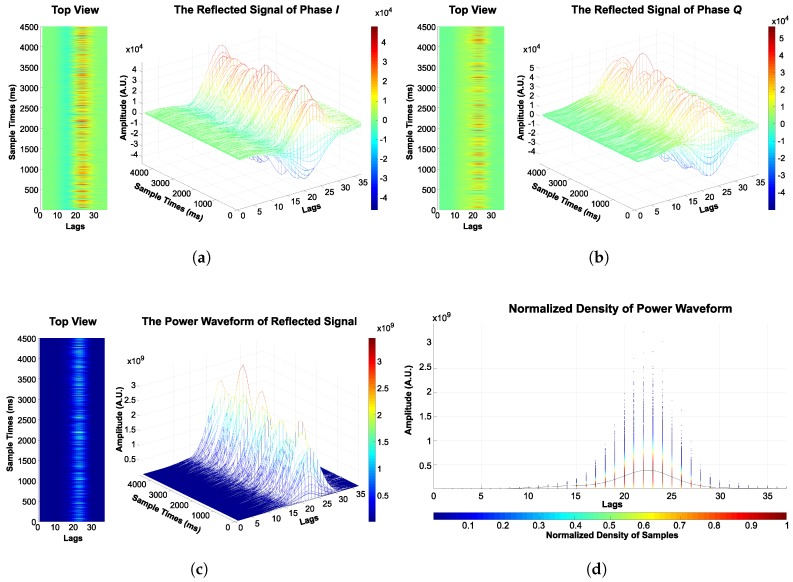
An example of a real reflected signal from the BeiDou G4 satellite captured on 3 January 2014: (**a**) phase *I*; (**b**) phase *Q*; (**c**) integration of phases *I* and *Q* ($I^{2}+Q^{2}$) and (**d**) normalized density of the sample.

**Figure 7:**
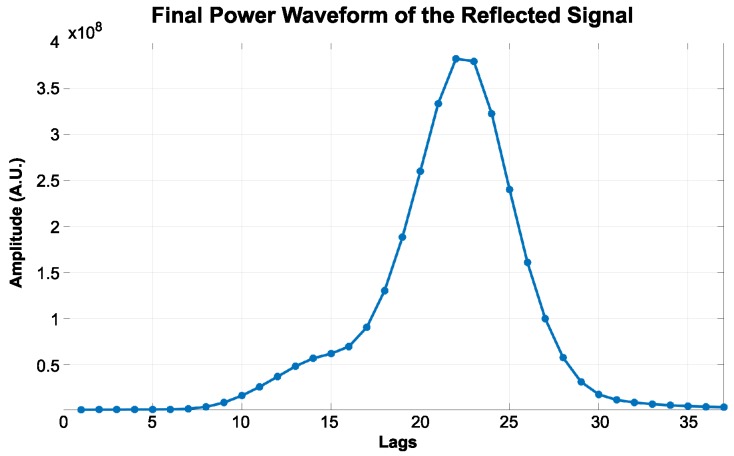
The final power waveform of an example of a real reflected signal from the BeiDou G4 satellite captured on 3 January 2014.

The reflected signal from the BeiDou G4 satellite was collected over the ocean surface at the same time as the in situ wind speed. The signal consisted of phase *I* and *Q* components, examples of which are presented in Figure 6a,b, respectively. Subsequently, the *I* and *Q* components were integrated by $I^{2}+Q^{2}$ in order to compute the power waveform, as shown in Figure 6c. The scatter plot in Figure 6d shows the normalized density of each lag, and the black line shows the connection of the mean values between lags. Accordingly, the final power waveform is shown in Figure 7.

**Figure 8:**
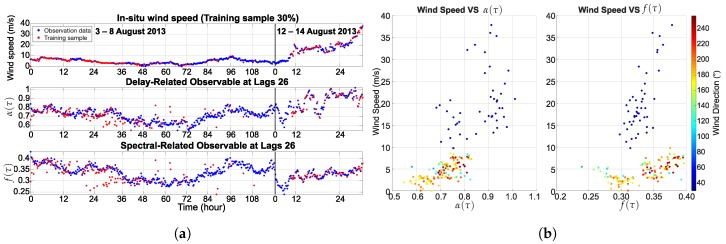
An example of the reflected signal at lag 26: (**a**) 30% training sample; (**b**) relationship between the training sample and the wind speed.

**Figure 9:**
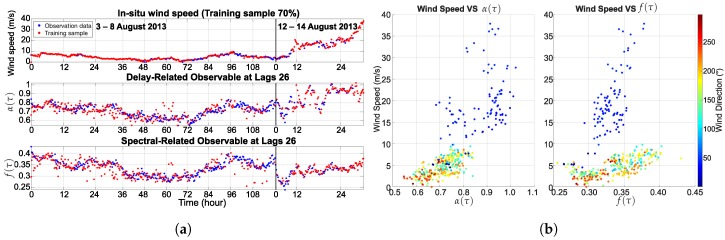
An example of the reflected signal at lag 26: (**a**) 70% training sample; (**b**) relationship between the training sample and the wind speed.

The sensitivity of the power waveform to wind speed is a key parameter for wind speed estimation. This research used the delay-related and spectral-related observables, computed from the power waveform, as input information for the ANN approach. The delay-related observable is expressed by:

(20) $$\alpha \left(\tau \right)=\frac{Z_{r}\left(\tau \right)-N_{floor}}{Z_{r}^{max}\left(\tau \right)-N_{floor}},$$

where $Z_{r}\left(\tau \right)$ is a delay, and $N_{floor}$ is the noise floor of the power waveform. $Z_{r}^{max}$ is the peak amplitude, which is computed by a third-degree polynomial on the power waveform. The spectral-related observable is expressed by:

(21) $$f\left(\tau \right)=\frac{\int_{f_{1}}^{f_{2}}f\cdot S_{r}\left(f,\tau \right)df}{\int_{f_{1}}^{f_{2}}S_{r}\left(f,\tau \right)df},$$

where $S_{r}\left(f,\tau \right)$ is the Doppler spectrum, and $f_{1}$ to $f_{2}$ is the frequency range of the Doppler spectrum. The cross-validation function of MATLAB 2016a based on HoldOut was used to select the training samples at 30% and 70% of all datasets, respectively. Figure 8 and Figure 9 present the training sample for the learning process of ANN, which consists of the in situ wind speed, the delay-related and spectral-related observables of BeiDou G4. In addition, the correlation of the reflected signal and the training sample are presented.

**Figure 10:**
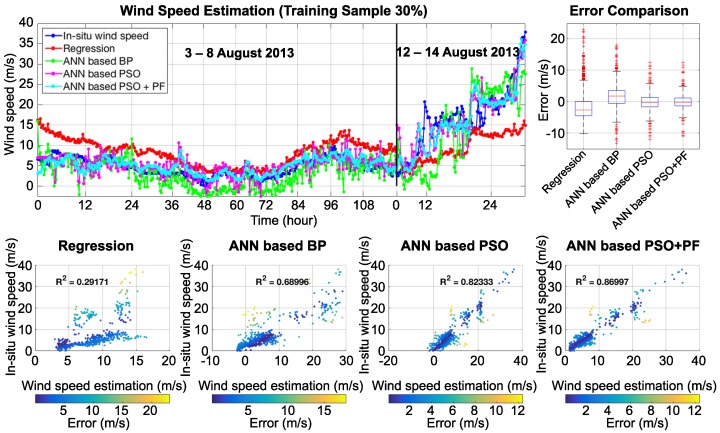
Comparison of wind speed estimation results using different techniques with 30% of the training sample.

**Figure 11:**
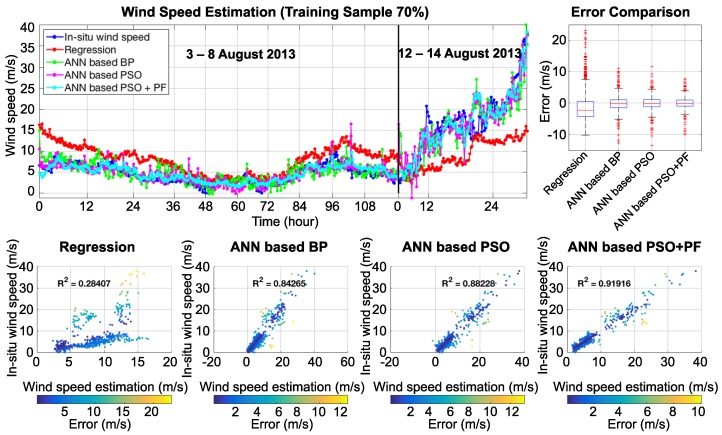
Comparison of wind speed estimation results using different techniques with 70% of the training sample.

Based on the training sample, it is evident that the derived results show good agreement with in situ wind speed measurement. Figure 10 and Figure 11 show the wind speed estimation results, which are presented in three different types of graphs: a line graph, a box plot, and a scatter plot. On the line graph, the proposed technique (cyan line) shows high efficiency and good correlation compared to a regression (red line), an ANN based back-propagation (BP, green line), and an ANN based PSO (magenta line) technique. The box plot presents the statistical error comparison of each technique. The integration of ANN based PSO + PF shows the lowest error and has a median value that is close to 0. The scatter plot presents the correlation of in situ wind speed with wind speed estimation. The first case, in Figure 10, shows the training sample at 30%. The R-square of the regression technique, the ANN based BP, the ANN based PSO, and the ANN based PSO + PF are 0.297, 0.689, 0.823, and 0.874, respectively. Figure 11 shows the second case: that with the training sample at 70%. The R-square of regression technique, the ANN based BP, the ANN based PSO, and the ANN based PSO + PF are 0.284, 0.842, 0.882, and 0.913, respectively. The covariance matrices of the particle filter were set by a trial and error technique. A comparison of the statistical results is illustrated in Table 1.

## 5. Discussion

In order to assess the efficiency of the proposed technique, the root mean square error (RMSE) was used as a standard statistical metric to measure the model’s performance. The RMSE used for evaluation in this section is expressed by:

(22) $$RMSE=\sqrt{\frac{1}{n}\sum_{i=1}^{n}{\left(y_{i}-\hat{y_{i}}\right)}^{2}},$$

where $y_{i}$ is the in situ wind speed measurement from the buoy, $\hat{y_{i}}$ is the wind speed estimation, and *n* is number of observations. Table 1 shows the statistical results of each technique. The overall RMSE of the proposed technique is better than the regression, the ANN based BP, and the ANN based PSO techniques by approximately 4, 1.5, and 0.5 m/s, according to the PF based autoregressive model that used information from the previous time step. For wind speeds higher than 20 m/s, the results showed greater errors compared to lower wind speeds due to the lack of high wind speed samples. The in situ data used in this research only provided 18 samples for wind speeds higher than 20 m/s. In order to improve the efficiency of the high wind speed results, an appropriate number of training samples is required. For this research, when the cross-validation function was applied for selecting 30% of the training sample, the total random sample is 174 samples, which consist of 156 samples of wind speed higher than 20 m/s and 18 samples of wind speed lower than 20 m/s. Likewise, when 70 % of the training sample was selected, the total random sample is 385 samples, which consist of 350 samples of wind speed higher than 20 m/s and 35 samples of wind speed lower than 20 m/s.

**Figure 12:**
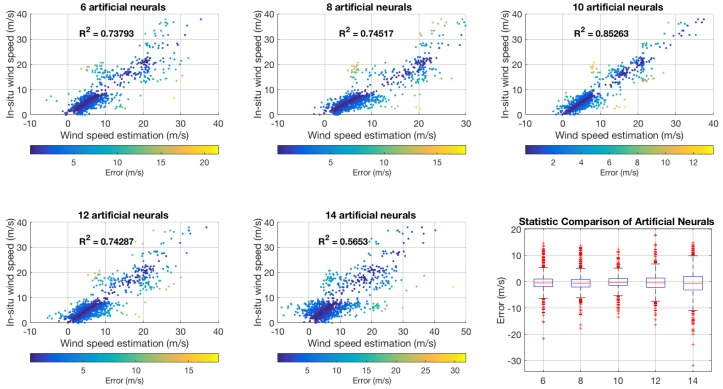
Comparison of wind speed estimation using ANN based PSO for different hidden layers.

The number of artificial neurons in the hidden layers is an important factor when deciding to select an ANN architecture. This research compared different numbers of neurons (6, 8, 10, 12, and 14 neurons) in order to find the optimal number of artificial neurons for wind speed estimation. Figure 12 shows the scatter plots of the relationship of in situ wind speed measurements and wind speed estimation for the different hidden layer architectures. These combine the results of the assessments using 30% and 70% of the training dataset and include $R^{2}$ values. The statistical comparison of each hidden layer is represented in the box plot.

The overall RMSE of the ANN based PSO with 10 artificial neurons was approximately 2.9 m/s when 30% of the training sample was used and 2.3 m/s when 70% of the training sample was used, as shown in Table 2. According to these results, the optimal number of neurons for wind speed estimation from the information of the BeiDou reflected signal is 10 neurons, since this produced the lowest RMSE.

**Figure 13:**
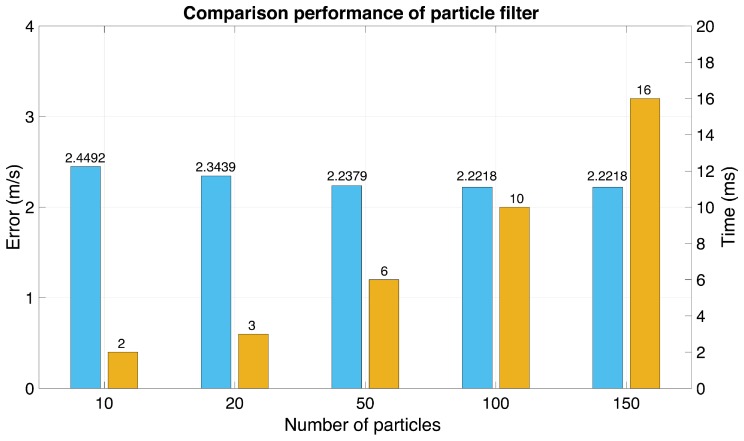
Comparison of the root mean square error (RMSE) (cyan) with the computation time (orange) of the particle filter.

One important factor for achieving a higher efficiency of particle filters is the number of particles. The filter approximates the posterior distribution with high efficiency when the number of particles tends to infinity. On the other hand, the computational cost grows with the number of particles. For this reason, this research compared different numbers of particles in order to find the optimal number for wind speed estimation, as presented in Figure 13.

The RMSE results shown in Figure 13 are the average value from the 30% and 70% training samples with a computation time of one iteration. With 50 particles, the RMSE was approximately the same as for 100 and 150 particles, but less computation time was required.

**Figure 14:**
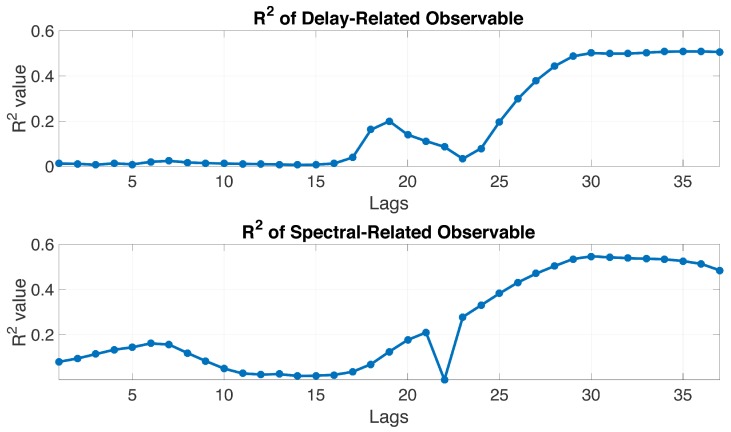
$R^{2}$ values of the delay-related and spectral-related observables.

The linear regression model was used to compute the $R^{2}$ value of each lag for the delay-related and spectral-related observables. According to Figure 14, the $R^{2}$ values of data collected from the two periods of time increased after the peak of the final power waveform. As a result, this research selected the data from lags 26 to 37 as the input information for the ANN approach.

**Figure 15:**
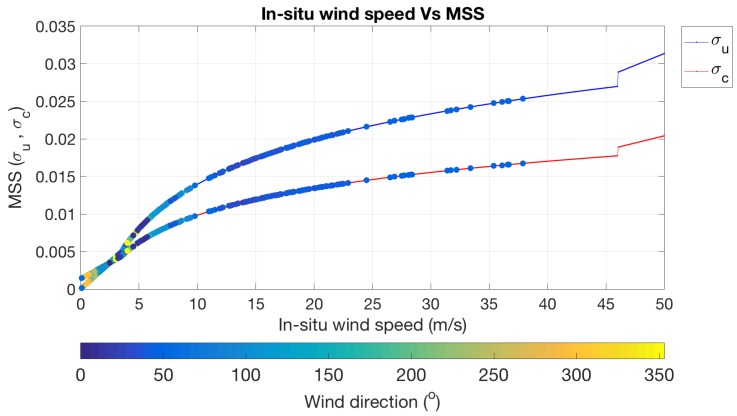
The relationship of in situ wind speed measurement and mean square slope. The up and cross wind speed are represented in blue and red solid line, respectively. The colored points represent up and cross wind speed result from our case study.

The relationship of in situ wind speed measurement and mean square slope is shown in Figure 15. The function of the sea surface roughness model for computing the up and cross wind speed in Equation (9) will depend on three conditions as shown in Equation (10). For the first condition, the result of wind speed calculated from the model is related to in situ wind speed measurement. For the second condition, the result will change with logarithm function. Finally, for the third condition, the result will change with static gain.

## 6. Conclusions

Currently, the new remote sensing technology of GNSS-reflectometry has shown benefits for specific targeted measurements. Hence, the reflected signal information for ocean wind speed was studied. The regression technique is useful and easier for wind speed estimation by fitting in situ wind speed measurements. On the other hand, it does not learn the characteristics of variables. The main idea of this research was to apply the artificial neural network combined with the particle filter for wind speed estimation based on the reflected signals from the BeiDou G4 satellite. Moreover, particle swarm optimization was implemented to solve for the weights and biases of the artificial neural network. From the two periods of dataset observation, 3 to 8 and 12 to 14 August 2013, several available signals of BeiDou GEO were used. In particular, for the period from 12 to 14 August 2013, the data was collected during the typhoon Utor. The experimental results show the effectiveness of the proposed technique by comparing it with the in situ wind speed measurements from a buoy. The overall RMSEs of ANN combined with PF were 2.4997 and 1.9758 m/s for the 30% and 70% training samples, respectively. The results of the ANN based PSO approach shows high efficiency for learning characteristics of the reflected signal for wind speed estimation. Using the performance of the PF approach with ANN enhances the effectiveness of estimating wind speed especially for time series observation. Future work will involve the integration of the reflected signal information from other satellite systems, such as GPS, GLONASS and Galileo, in order to achieve greater efficiency.

## Figures and Tables

**Table 1:** Comparison of the statistical results of wind speed estimation.

Algorithm	RMSE (m/s)					
	30%			70%		
	WS < 20	WS > 20	Overall	WS < 20	WS > 20	Overall
Regression	4.7839	13.4543	5.9009	4.7261	13.6445	5.8908
ANN based BP	4.4942	6.5375	4.6796	2.5595	4.4798	2.7516
ANN based PSO	2.8843	3.4397	2.9299	2.3081	3.1553	2.3826
ANN based PSO combined with PF	2.3982	3.5124	2.4997	1.8524	3.1126	1.9758

**Table 2:** Comparison of the statistical results relative to the number of neurons in the neural network.

Number of Neurons	RMSE (m/s)					
	30%			70%		
	WS < 20	WS > 20	Overall	WS < 20	WS > 20	Overall
6	3.5606	5.9404	3.7926	3.1049	5.5643	3.3540
8	3.5511	5.8782	3.7771	3.0445	5.2631	3.2650
10	2.8843	3.4397	2.9299	2.3084	3.1553	2.3828
12	3.3689	6.6578	3.7200	3.3045	4.8027	3.4404
14	5.2888	5.5649	5.3101	4.9382	5.6721	4.9974
